# Patient perspectives on the need for implanted device information: Implications for a post‐procedural communication framework

**DOI:** 10.1111/hex.13273

**Published:** 2021-05-11

**Authors:** Natalia A. Wilson, Amanda J. Reich, Jove Graham, Deepak L. Bhatt, Louis L. Nguyen, Joel S. Weissman

**Affiliations:** ^1^ College of Health Solutions Arizona State University Phoenix AZ USA; ^2^ Center for Surgery and Public Health Brigham and Women's Hospital Boston MA USA; ^3^ Geisinger Danville PA USA; ^4^ Heart and Vascular Center Brigham and Women's Hospital Boston MA USA; ^5^ Division of Vascular and Endovascular Surgery Brigham and Women's Hospital Boston MA USA

**Keywords:** implanted devices, patient perspective, patient‐centered communication, post‐procedural communication, shared decision making, unique device identifier

## Abstract

**Background:**

Shared decision making and patient‐centred communication have become part of pre‐procedural decisions and perioperative care across medical specialties. However, gaps exist in patient communication about the implanted device received and the benefits in sharing information about their procedure and device.

**Objective:**

To understand the patients' knowledge of identifying information for their implanted devices and perspectives on sharing their implanted device information.

**Methods:**

Four focus groups were conducted with patients who had received a cardiac or vascular implanted device from one of the study sites within the previous 6 months. Data were transcribed and thematically analysed.

**Results:**

Five themes emerged: lack of awareness of identifying information on implanted devices; value of information on implanted devices; varying trust with sharing device information; perceived risk with sharing device information; and lack of consensus on a systematic process for tracking implanted devices.

**Discussion:**

Patients desire post‐procedural information on their implanted device and a designated plan for longitudinal follow‐up, but lack trust and perceive risk with broadly sharing their implanted device information.

**Conclusion:**

After receiving an implanted device, post‐procedural patient communication needs to be expanded to include identifying information on the device including the unique device identifier, how long‐term tracking will be supported and the process for notification in case of a problem with the device. This communication should also include education on how sharing device information supports patients' long‐term health care, post‐market safety surveillance and research.

**Patient or Public Contribution:**

The research team included members who were also patients with implanted devices.

## 1 INTRODUCTION

Implanted devices are an important part of medical care that address patient morbidity and mortality and augment patient quality of life. These devices that are intended to be placed in a surgically or naturally formed cavity of the human body^1^ are very common in procedures across medical specialties. For example, an estimated seven million Americans are living with a hip or knee replacement.^2^ Over 500 000 procedures involving a pacemaker or cardioverter/defibrillator were performed in the United States in 2014.^3^ Approximately 480 000 inpatient percutaneous coronary interventions were performed in the United States in 2014, most involving implantation of one or more cardiac stents.^4^

Despite the benefits for patients, problems occur with medical devices over their lifespan that may impact patient health, well‐being and safety. Examples include the high profile recalls of cardioverter/defibrillator leads^5^ and metal‐on‐metal hip implants,^6^ complications with surgical mesh used in urogynaecologic procedures^7^ and the Essure device,^8^ association of breast implants with anaplastic large cell lymphoma,^9^ increased risks of stent thrombosis with first‐generation drug‐eluting stents^10^ and a potential mortality risk associated with paclitaxel‐containing device use.^11^, ^12^

Shared decision making and patient‐centred communication have increasingly become part of pre‐procedural decisions and perioperative care across medical specialties.^13^, ^14^, ^15^ They foster exchange of information relevant to patients and patients' involvement in their health care, decisions, self‐management and satisfaction.^16^, ^17^, ^18^ Frameworks and checklists have helped support the patient pre‐ and post‐procedure and with the transition from the health‐care system at discharge, with much of this focus on recovery and follow‐up.^19^, ^20^, ^21^ However, gaps exist in communication surrounding the implanted device a patient receives. Lacking is a standard method for all patients to receive identifying information including the unique device identifier (UDI) on their implanted device, communication about the process that will be followed if a problem with their implanted device (eg safety alert, recall) arises in the future, and in some cases detailed information on the functioning of the device they have received.^22^ In addition, communication to patients is scant regarding how sharing of information about their procedure and implanted device benefits their long‐term health care through device tracking, post‐market safety surveillance and research.^23^, ^24^

This work was part of a larger study (*UDI2Claims*) to develop a process to transmit UDIs for devices implanted in patients during procedures to insurance claims.^25^, ^26^ As part of the United States Food and Drug Administration's framework to advance medical device safety, a UDI system was established through issuance of the Unique Device Identification System Rule in 2013. The Rule mandates manufacturers to include UDIs on the labels and packaging of their marketed devices.^27^ (components of the UDI are a device identifier (DI) that identifies the manufacturer and model and the production identifier (PI) that identifies, as available, the lot number, serial number and expiration date^28^). The UDI can be scanned during a patient procedure and documented in the patient's electronic health record where it is available for multiple uses such as sharing with the patient, implant identification in clinical care and aggregation with other device data for post‐market safety surveillance and research.^29^ The European Union has also mandated a UDI System through its Medical Device Regulations with dates of compliance starting in 2021.^30^ The aim of the work reported here was to answer the following research questions: (a) Are patients aware of identifying information for their implanted device including the UDI? (b) Do patients want information on their implanted device shared? And (c) what are the critical influences that guide their view?

## 2 METHODS

### 2.1 Settings and participant selection

The study settings were two hospitals in the United States, Brigham and Women's Hospital (BWH) and Geisinger Health (GH), as part of the larger *UDI2Claims* study. Four focus groups were conducted, with one at each site in June 2017 and again in April 2019. Purposive sampling was utilized. Criterion for inclusion in a focus group was patients who had received a cardiac or vascular implanted device at BWH or GH in the 6 months prior to the focus group. Lists of patients meeting this criterion were generated by clinical staff at BWH and GH through evaluation of electronic health records.

Potential participants from BWH and GH were contacted and invited to participate in a ninety‐minute focus group. Patients who agreed to participate received a phone reminder a few days prior to the focus group date. The study was approved by the Partners Office of Research Integrity and Assurance.

### 2.2 Guide and data collection

Domains and questions in the focus group guide were informed by the literature, input by research team members experienced in device work and qualitative research, patient research partners and members of the *UDI2Claims* expert advisory panel of stakeholders, which included researchers, clinically active physicians, insurers, representatives of hospital supply chain management, information technology, and administration and representation from the United States Food and Drug Administration. The focus group guide domains included Information, Responsibility, Access, Use and Notification. Questions for the focus groups were refined after 2017 in order to achieve greater focus on patients' understanding and preferences related to device identification and information sharing. Patients in the 2017 focus groups included important perceptions on their experiences with medical care; however, this transcended the project focus on implanted device identification, UDI and sharing this information (focus group guides available in Appendices A & B).

Prior to commencement of the focus groups, participants completed a registration questionnaire and were orally consented. The questionnaire asked about information on their device and listed a number of possible concerns, one example being ‘No one is tracking the device nationally to find out problems’. Responses used a 4‐point Likert scale ranging from 1 (very worrisome), 2 (somewhat worrisome), 3 (a little worrisome) to 4 (not worrisome at all). One researcher led and facilitated each focus group using the focus group guide developed by the research team. One to two additional research team members were present at focus groups to take notes. Upon completion of the focus group sessions, participants were provided information and encouraged to contact researchers with any further thoughts or perceptions they wished to share. Audio recordings of the focus group sessions were obtained and transcribed.

### 2.3 Data analysis

Four focus groups were scheduled, and it was determined after preliminary analysis that data saturation was adequate and further sampling was not necessary. Transcribed audio recordings were analysed utilizing a multi‐step process, guided by Ritchie and Lewis' Framework Technique.^31^ Two members of the research team experienced in qualitative research methods independently familiarized themselves with the data by reviewing the transcripts and created a list of inductive codes. The researchers compared their independent findings and agreed upon a thematic framework. This was then applied to the transcripts. Key data for each theme were obtained, and representative quotes were identified. Through an iterative process, disagreements were adjudicated, and a consensus was reached on major themes and subthemes from the focus groups. Data management was manual.

Descriptive statistics were used to analyse the registration questionnaire. Data were aggregated and organized using Microsoft Excel.

## 3 RESULTS

Twenty‐four patients participated in the four focus groups. A majority were over the age of 65 (58%), female (54%) and had at least some college education (66%). Ninety‐five percentage of participants reported White race. For insurance status, a mix of Medicare and private insurance coverage was reported (Table 1).

**TABLE 1:** Participant demographics

	Number (n = 24)	%
Age		
65 and younger	10	41.7
66‐75	9	37.5
75+	5	20.8
Sex		
Male	11	45.8
Female	13	54.2
Education		
Some high school but did not graduate	2	9.5
High school graduate or GED	5	23.8
Some college^a^	14	66.7
No reply	3	—
Ethnicity		
Hispanic/Latino	0	0
Not Hispanic/Latino	22	100
No reply	2	—
Race		
White	21	95.5
Black or African American	0	0
Asian	0	0
Native Hawaiian or Pacific Islander	0	0
American Indian or Alaska Native	0	0
Other	1	4.5
No reply	2	—
Insurance		
Medicare	6	27.3
Medicaid	0	0
Private	7	31.8
Medicare and private	5	22.7
Medicare and other	2	9.1
Medicare, Medicaid, Private	2	9.1
No reply	2	—

^a^ Some participants may have a college or graduate degree. In 2019, we added those as options, so for consistency purposes, we are using the 2017 Education ‘Some college’ for anyone that put college, regardless of whether or not they may have a degree.

In terms of patient concerns regarding their implanted device, on average, patients reported ‘a little worrisome’ (3.1‐3.4). This included ‘no one is tracking the device nationally to find out problems’ (Table 2).

**TABLE 2:** Patient concerns regarding implanted device

Concern	Mean response^a^ (n = 24)
I will need to replace the device if there is a problem	3.1
If I move, my doctor won't know what happens to me	3.4
The device could be recalled, and I wouldn't know it	3.4
No one is tracking the device nationally to find out problems	3.1
I can't afford it	3.1

^a^ Patients' concerns regarding their implanted device, using a 4‐point Likert scale ranging from 1 (very worrisome), 2 (somewhat worrisome), 3 (a little worrisome) to 4 (not worrisome at all).

Five key themes with associated subthemes emerged across the focus groups: (a) lack of awareness of identifying information on implanted devices, (b) information on implanted devices is valuable, (c) varying trust in sharing device information, (d) perceived risks with sharing device information and (e) lack of consensus on a systematic process to track implanted devices (Table 3).

**TABLE 3:** Patient perspectives on implanted device information: themes and subthemes

Theme	Subtheme
Lack of awareness of identifying information on implanted devices	Did not know about unique device identifiers (UDIs) Did not routinely receive an implant card Unclear who may already receive their procedure or device information
Information on implanted devices is valuable	Benefit for clinical care—implant identification Lessens potential health risk Benefit through research
Varying trust in sharing device information	Trust hospital, physicians, self with information Mistrust insurance companies, government Mixed trust of manufacturers, national database/registry
Perceived risks with sharing device information	Denial of insurance coverage Use not in patient's best interest Potential loss of privacy
Lack of consensus on a systemic process to track implanted devices	Physicians, Hospital, Manufacturer Mixed government Unclear of overall system

### 3.1 Theme 1: Lack of awareness of identifying information on implanted devices

Participants overall lacked details on their implanted devices and had a lack of clarity on what information was available to them. Whereas some patients had implant cards with information such as device manufacturer and model, others never received a card. No participants were aware of the availability of UDIs. Two patients discovered during the focus groups that their implant cards contained the UDI.

I did not know about (UDI), but it did not surprise me (to know devices can be identified this way), in this day and age

Other participants described a lack of engagement with providers when they received their implanted devices and a lack of knowledge that information on an implant card was even available for patients. There was the general sentiment by participants that identifying information on their implanted device was documented somewhere within the health‐care system and could be accessed as needed.

(My) husband asked why I did not get a card. Made me think why I do not have one

I never gave a thought to ask for a card

### 3.2 Theme 2: Information on implanted devices is valuable

Patients endorsed the value of having identifying information including the UDI for their implanted devices available. Subthemes included the benefit for their clinical care and reducing patient health risk. They acknowledged that unique device identifiers are important if a patient moves or existing relationships are no longer available.

I am thinking this will be useful down the road. I may be moving to California or Arizona

The physician who put in my device was gone after six months of putting it in. I never heard from him again

Others noted that device tracking was beneficial beyond their own personal concerns, describing important implications of being able to track devices, link to patients and have this information available for broader use. A subtheme was the benefit in research, which could help other patients.

I like research, to help learn about the possibility of creating better health

### 3.3 Theme 3: Varying trust in sharing device information

Patients felt most comfortable with sharing their implanted device information within the medical profession—providers who would care for them and the hospital in which they would receive care.

(I) have no problem with people knowing in the medical system

Most patients also felt they should have their own information.

(You) need to be your own patient advocate

Most were not interested in sharing information with the insurance company or the federal government. Trust of manufacturers was mixed, and participants overall were unclear whether manufacturers kept records of their implanted device.

I cannot imagine why they (the insurance company) would want it. I would not give it to them

Why does this have to be involved with the government when tracking devices for health?

I have some concern about (manufacturers) covering up a problem

### 3.4 Theme 4: Perceived risks with sharing device information

Patients perceived risk in sharing their implanted device information with insurance providers and the federal government. Subthemes included denial of insurance coverage, use that was not in their best interest and loss of privacy.

Some patients described concern that insurance providers might use the information about their implanted device against them.

What are the insurance companies going to do if they have (device identifying information)‐be on the patient side or not?

Other patients expanded on this concern, although a participant in one focus group did inform others during discussion that procedural data are already shared with insurance providers for payment purposes.

(I) worry if the insurance company knows I had a stent put in 10 years ago and had a couple more put in now. Will that be used against people?…What about some 30‐year olds? Are they going to lose their health insurance because the insurance company finds out about some of these medical issues through a database? This guy has a bunch of stents in him. We need to find a reason to get rid of him because he costs hundreds of thousands of dollars.

Will insurance not take me if I have thirty‐five implants?

Patients expressed general unease with the federal government having their information, although a participant in one focus group did inform others that the federal government has an enforcement responsibility where they need data, for example for recalls. If there is an issue, they may need to step in.

People do not have faith in the government

### 3.5 Theme 5: Lack of consensus on a systemic process to track implanted devices

When patients were asked about what process they would like implemented to track devices, and who should be responsible, there was very little consensus across participants. For example, both clinical providers and manufacturers were identified as possible responsible parties. While they did not agree on structure and logistics, patients overall expressed surprise and frustration that no comprehensive system currently exists. As illustrated by one patient,

I don't know how many millions & millions of cars are sold in this country every year. I have a 17 year old Dodge pickup truck that I just got a recall notice. I did not buy this new. I did not buy it from a dealer, but they know I have that car. (A) 17 year old car that has a recall… If they can track a 17 year old car I bought from a private party, why can they not do that with (cardiac) stents?

Other patients expressed the sentiment that ‘this should have been figured out by now’ and noted that the data should be centralized somehow.

Somebody has to have all the knowledge

Patients desired a systemic way for implanted device information to be stored and a clear method to be notified as a patient if there were implant problems, but there was a lack of consensus on how/who should do this: medical providers, manufacturers, the United States Food and Drug Administration and/or a national registry.

## 4 DISCUSSION

The focus groups uncovered patient perceptions, concerns and contextualized issues about their implanted device information. Patients felt that availability of their implanted device information including the UDI was valuable for their individual health care and for the greater good when used in device tracking and for research. They desired a systemic process for their implanted device information to be stored and a clear plan for longitudinal follow‐up and notification in cases of device problems. Also uncovered in this study are as follows: (a) there is a lack of standard practice for patients to receive identifying information including the UDI for their implanted devices, (b) patients perceive risk with their information being shared beyond personal use or within the health‐care system, and (c) critical influences that guide their views are trust, benefit in use and protection of privacy and security.

*There is a lack of standard practice for patients to receive information on their implanted device*. Not all patient participants in the study had received implant cards. Patients were not proactive in requesting their implanted device information and not only had a lack of clarity about what information was available for them but also seemed to rely on their implanted device information including the UDI being easily available somewhere in the health‐care system. These outcomes are in conflict with an important goal of patient‐centred communication, to facilitate patient participation in their care.^18^ Further, shared decision‐making encounters may be both incremental and iterative.^32^ Patient preferences remain relevant after implantation of a device, especially due to the longitudinal course of implanted devices. Patients may need replacement of their implanted device due to its natural ageing or a problem. Whatever the scenario, understanding the patient experience, having all relevant device identifying information available and utilizing shared decision making for next steps are highly relevant.

*Patients perceive risk with their information being shared beyond personal use or the health‐care system*. Patients' interest in sharing their device information was dependent on with whom it was being shared and for what purposes. Participants were supportive of sharing their device data within the health‐care system for health‐care decision making and for purposes of research. They were concerned with sharing their information with insurance companies and the federal government. However, they seemed unaware that insurance companies may already receive their procedure or device information and that the United States Food and Drug Administration, a federal government agency, has responsibility for medical device oversight. Sentiment softened some as participants became aware during the focus groups of uses of their information beyond the hospital system, including for implanted device tracking and research, and the value for patients' long‐term health care.

*Critical influences that guide their views are trust, benefit in use* and *protection of privacy and security*. Our findings on implanted devices were consistent with existing literature regarding patient data more generally, which indicates that most patients are supportive of sharing data for health‐care decision making and for research, but with conditions that foster trust, privacy, data responsibility and purpose.^33^, ^34^, ^35^, ^36^ Patients are motivated to advance health‐care knowledge and support their own care and that of future patients.^37^ Also consistent with existing literature is weakened trust between patients and insurance companies and between patients and the federal government. Patients do not trust that these stakeholders will act in their best interest or properly protect their privacy.^36^, ^38^, ^39^ Patients express preferences for sharing their implanted device information with clinical providers over insurers and government agencies. Patient willingness to share data will be contingent upon addressing and mitigating their perceptions of risk.

Study limitations include that the focus groups were small and were attended by White, mostly older participants. Limited types of implanted devices were represented with a focus on cardiac and vascular surgery‐implanted devices. Focus group participants were recruited from only two hospital sites in the United States, which may have narrowed the patient experiences and perceptions captured.

## 5 CONCLUSION

Results from the focus groups indicate that patients overall lack information about their implanted device, are not aware of the availability of UDIs for documentation and tracking, and are unaware of a designated plan for longitudinal follow‐up of their device in case of problems. This is discordant with goals of shared decision making and patient‐centred communication.

Results also indicate that patients perceive risk with sharing their information with insurance companies, despite procedural information already being shared and the potential benefit for patients' long‐term health care, device post‐market safety surveillance and research by augmenting already shared information. Patients also perceive risk in sharing information with the federal government despite the United States Food and Drug Administration's responsibility for medical device safety and oversight. This is an important insight that implies a lack of patient knowledge of reasons and benefit in data sharing and with whom their data are already being shared.

Based on the findings of this study and within the context of shared decision making and patient‐centred communication, recommendations for clinical improvement include the following:

1. Expand post‐procedural patient communication to consistently include information on patients' implanted devices including the UDI, how long‐term tracking will be supported and the process for notification of the patient in case of an implanted device problem.
2. Ensure that all patients receive a wallet card or e‐version of their implant information including the UDI. A standard model could be developed from the current card for a breast implant, implantable defibrillator or on‐going international efforts.^40^
3. Clarify key area(s) where patients' implanted device information is documented (eg electronic health record) and where it is shared for device tracking, post‐market safety surveillance and research (eg specific clinical registry, United States Food and Drug Administration).
4. Provide education on how device information sharing supports patients' long‐term care, post‐market safety surveillance and research. The National Breast Implant Registry Patient FAQs document could be adapted as it directly addresses the use of the collected information and how participation can benefit patients.^41^
5. Review information provided during post‐procedural patient communication in the first post‐discharge office visit.

Additionally, it is recommended that future research studies be done to augment these findings, particularly by engaging patients who received implanted devices in a broader array of procedures and medical specialty areas and engaging patients who self‐identify as members of minority or underserved communities. Lastly, given the observation that some focus group members did not receive an implant card and were unaware that cards and UDIs were available, this is an important area for a focused quality improvement project at implanting facilities.

## 6 IMPLICATIONS FOR PATIENTS AND PRACTICE

Implementation of these recommendations will promote improved communication and understanding between patients who receive implanted devices and their providers. They support not only patients being more knowledgeable about their health care but also being engaged participants in advancing medical device safety for themselves and others. These recommendations additionally foster improved documentation of implanted devices via the UDI during the procedure, so standard information is available for patients and is available to support greater longitudinal tracking of devices and availability of data for post‐market safety surveillance and research. Understanding areas that patients perceive as risks in device data sharing will inform development of a standardized system that is inclusive of education and responsive to patient preferences. Further research and quality improvement projects can fine‐tune these areas and support greater generalizability. In this way, shared decision making and patient‐centred communication methods will augment care and safety for patients who receive and live with implanted devices.

## CONFLICT OF INTEREST

Natalia Wilson purchased stock options in VitreosHealth; and has received partial funding for the BUILD project from Johnson & Johnson and Medtronic. Deepak Bhatt served as a member of the advisory board for Cardax, CellProthera, Cereno Scientific, Elsevier Practice Update Cardiology, Level Ex, Medscape Cardiology, MyoKardia, PhaseBio, PLx Pharma and Regado Biosciences; a member of the Board of Directors for Boston VA Research Institute, Society of Cardiovascular Patient Care and TobeSoft; a chair for American Heart Association Quality Oversight Committee; a member of Data Monitoring Committees for Baim Institute for Clinical Research (formerly Harvard Clinical Research Institute, for the PORTICO trial, funded by St. Jude Medical, now Abbott), Cleveland Clinic (including for the ExCEED trial, funded by Edwards), Contego Medical (Chair, PERFORMANCE 2), Duke Clinical Research Institute, Mayo Clinic, Mount Sinai School of Medicine (for the ENVISAGE trial, funded by Daiichi Sankyo) and Population Health Research Institute; has received honoraria from American College of Cardiology (Senior Associate Editor, Clinical Trials and News, ACC.org; Vice‐Chair, ACC Accreditation Committee), Baim Institute for Clinical Research (formerly Harvard Clinical Research Institute; RE‐DUAL PCI clinical trial steering committee funded by Boehringer Ingelheim; AEGIS‐II executive committee funded by CSL Behring), Belvoir Publications (Editor‐in‐Chief, Harvard Heart Letter), Canadian Medical and Surgical Knowledge Translation Research Group (clinical trial steering committees), Duke Clinical Research Institute (clinical trial steering committees, including for the PRONOUNCE trial, funded by Ferring Pharmaceuticals), HMP Global (Editor‐in‐Chief, Journal of Invasive Cardiology), Journal of the American College of Cardiology (Guest Editor; Associate Editor), K2P (Co‐Chair, interdisciplinary curriculum), Level Ex, Medtelligence/ReachMD (CME steering committees), MJH Life Sciences, Population Health Research Institute (for the COMPASS operations committee, publications committee, steering committee and USA national co‐leader, funded by Bayer), Slack Publications (Chief Medical Editor, Cardiology Today's Intervention), Society of Cardiovascular Patient Care (Secretary/Treasurer), WebMD (CME steering committees) and others (Clinical Cardiology (Deputy Editor), NCDR‐ACTION Registry Steering Committee (Chair) and VA CART Research and Publications Committee (Chair)); has received research funding from Abbott, Afimmune, Amarin, Amgen, AstraZeneca, Bayer, Boehringer Ingelheim, Bristol‐Myers Squibb, Cardax, Chiesi, CSL Behring, Eisai, Ethicon, Ferring Pharmaceuticals, Forest Laboratories, Fractyl, Idorsia, Ironwood, Ischemix, Lexicon, Lilly, Medtronic, MyoKardia, Pfizer, PhaseBio, PLx Pharma, Regeneron, Roche, Sanofi‐Aventis, Synaptic and The Medicines Company; has received royalties from Elsevier (Editor, Cardiovascular Intervention: A Companion to Braunwald's Heart Disease); served as a site co‐investigator for Biotronik, Boston Scientific, CSI, St. Jude Medical (now Abbott) and Svelte; served as a trustee for American College of Cardiology; and has received unfunded research grants from FlowCo, Merck, Novo Nordisk and Takeda. Amanda Reich, Jove Graham, Louis Nguyen and Joel Weissman declared no conflict of interest.

## AUTHOR CONTRIBUTION

Natalia Wilson was involved in study design, data acquisition, analysis and interpretation, and manuscript writing, review, editing and final version preparation. Amanda Reich was involved in study design, data acquisition, analysis and interpretation, project administration, and manuscript writing, review and editing. Jove Graham, Deepak Bhatt and Louis Nguyen supported data acquisition and critically reviewed and edited the manuscript. Joel Weissman as the principal investigator led study conceptualization, was involved in study design and data acquisition, and critically reviewed and edited the manuscript.

## Data Availability

The data that support the findings of this study are available on request from the corresponding author. The data are not publicly available due to privacy or ethical restrictions.

## APPENDIX A Focus Group Guide 2017

**Introduction (5 minutes)**

Good evening everyone. Thank you all for agreeing to participate in a focus group.

My name is _____ and this is ___________. We are researchers at Brigham and Women's Hospital. Today we would like to talk with you about your thoughts and opinions, as well as experiences with your implanted medical device. This focus group is a part of a research study that is about developing methods for storing information about your device in your electronic medical records.

As you may or may not know, implantable devices, like the ones you all have, now have unique device identifiers (UDIs). The UDI is a code for the manufacturer and model of your device, as well as other information like expiration date, lot number, or serial number. Right now that UDI may or may not be documented in your electronic medical record. We are interested in developing a system for the UDI to be documented in your medical record. The UDI will then be shared with your health insurance company, who then may or may not share it with a national registry or database. The goal of the registry or database, is to have information about lots of people's devices in one place so that the effectiveness and safety of the devices can be tracked and understood. This is a study that is funded by the Patient‐Centered Outcomes Research Institute.

Before we begin our discussion, I wanted to just cover a few ground rules for our discussion today.

1. There are no right or wrong answers today. Please make sure you are respectful of everyone's thoughts and opinions.
2. Please speak one at time so we make sure we can hear what everyone says.
3. Please keep confidential anything that was said in today's group.
4. Please feel free to say any names during our discussion. We will be audio recording our conversation today so we remember what everyone says. We will be deleting all names and places in the reports we write.
5. You do not have to answer any of the questions if you don't want to. If you think of anything you want to add after the group today, please contact us to share.
6. We will be ending our discussion today at xxpm. I might need to move us along at different points of the conversation to make sure we cover all of the topics.

Do you have any questions before we begin?

**Introductions** (10 minutes)

The first thing I would like to do is to have everyone introduce himself or herself and say how long they have had an implanted device.

**Initial thoughts** (10 minutes)

1. Did you know that your implanted medical device has a unique device identifier or UDI?
2. Do you know how to figure out what your UDI is?
3. Do you currently know your UDI or have some identifying information on your implanted device?
  - Do you have a card? If not, would you want a card?
4. Do you think it is important for you to know your UDI?
  - Why or why not?
  - If yes, when would you like to be given the UDI?
  - If yes, how would you like this information given to you?
5. How might knowing your UDI help you?
  - Have you had a situation where knowing your UDI would have been helpful? (Probe for: recalls of type of device, establishing with a new doctor)
  - Have you ever asked or needed to get your UDI from your doctor?
    - If so, how was that process?

**Storage of the information** (10 minutes)

Next we are going to talk about where your UDI is stored.

6. Who else do you think should have information about your UDI?
  a In other words, what doctors or medical providers do you think should have access to the information? Why?
  b Whose job do you think it is to know what your UDI is? (Probe for: doctor, hospital, insurance company)
7. Where do you think your UDI should be stored?
  a What are your thoughts about storing the UDI for your implanted device in your electronic medical record?
  b Is there anywhere else you think that this information should be stored?

**Sharing of the information** (20 minutes)

Next we are going to talk about your UDI being shared with different people and groups.

8. What are your initial thoughts about having the information about your implanted device shared with:
  a A national registry run by physician researchers
    i A patient registry is a system that collects data to look at outcomes for people with a specific disease, illness, or a particular device. The purpose of the registry is to study or track long‐term outcomes.
  b Somewhere in the federal government like the FDA
  c The device manufacturer
  d Your insurance company
9. What, if any concerns do you have with any of these groups having access to information about your device?
10. We would want to make sure that you feel confident that the information is stored and shared securely. What would help you be more comfortable with these groups having access to and using the information?
  a Information about how they are storing the data?
  b Information about how they are using the data?
  c For example:
    i The information would be used to help other patients like you.
    ii If the information would be used to help identify safety issues faster.

**Using the information** (15 minutes)

Information about your device could be used for multiple purposes. We are going to go through the different ways the information could be used and would like your feedback.

11. The first we will discuss is using stored UDIs for research purposes. (This information would be available to researchers through the national registry.)
  a What are your thoughts about your UDI being used in research?
  b What research would you like to see? In other words, what do you think are the important research topics or ideas you would like to see about your device?
12. The second way stored UDIs could be used is to let patients know if there is an issue with their device. For example, if the device is recalled.
  a How would you like to be notified if there is a problem with your device?
  b Who would you like to notify you if there was a problem? (Probe for: manufacturer, hospital, doctor, insurance company?)
13. The last purpose we are going to talk about for how stored UDIs could be used is to help your doctor provide care to you.
  a Examples of how a doctor might use this information to provide care include to know if you can use a MRI machine or to know what type of device you have in case of an emergency.
    i What information would you like ANY doctor who sees you to know about your device?
  b Your doctor might use information about your implanted device from research using the registry.
    i What would you want your doctors to learn about your implanted device from research using the registry?
    ii Who would you want to provide information to you on whether one device is more effective than another device? (Probe for: doctors, insurance company, FDA)

**Final Thoughts** (5 minutes)

14. Do you have any other comments you would like to share?

## APPENDIX B Focus Group Guide 2019

**Introduction** (5 minutes)

Good evening everyone. Thank you all for agreeing to participate in a focus group.

My name is ________, and this is _________ and ________. We are researchers at Brigham and Women's Hospital. This focus group is a part of a research study that is studying methods for recording and transferring information about implanted devices in electronic records and insurance claims. Today we would like to talk with you about your thoughts and opinions about this. The study is funded by the Patient‐Centered Outcomes Research Institute.

Before we begin our discussion, I want to cover a few ground rules for our discussion today.

1. There are no right or wrong answers. Please make sure you are respectful of everyone's thoughts and opinions.
2. Please speak one at time so we make sure we can hear what everyone says.
3. Please keep confidential anything that said in today's group.
4. Please feel free to say any names during our discussion. We will be audio recording our conversation so we remember what everyone says, but we will be deleting all names and places in the reports we write.
5. You do not have to answer any of the questions if you don't want to. If you think of anything you want to add after the group today, please contact us to share.
6. We will be ending our discussion today at 7:30 PM I might need to move us along at different points of the conversation to make sure we cover all of the topics.

**Do you have any questions before we begin?**

**[START TAPE RECORDERS]**

**A. PARTICIPANT INTRODUCTIONS (10 minutes)**

The first thing I would like is for everyone to introduce themselves. Please say your name and if you would like, tell us about your implanted device – what it's for and how long you have had it.

**B. UDI (5 minutes)**

The box in front of you is from an implanted device. This may not be the exact device you have implanted but is being used as an example. The barcode with the number below it is a unique device identifier or UDI. UDIs are now available for all implanted devices. When a patient has a device implanted the barcode can be scanned and the UDI can be recorded in the patient's electronic health record. The UDI is a code for the manufacturer and model of your device, as well as other information like expiration date, lot or serial number. This unique number for a medical device is similar to the VIN which is a unique identification number for your car.

Even though it is possible to scan the barcode and record the UDI in your electronic health record this is not being done at many hospitals. The UDI is also generally not listed on a patient's implant wallet card that they may receive after their procedure. That's why we are doing this study. We have been working on developing a system for the UDI to be recorded in your electronic health record and then shared with your health insurance company in the insurance claim form.

**B1. Do you have any questions about UDIs?**

**C. INITIAL THOUGHTS (10 minutes)**

We would like to start with getting some of your initial thoughts on the topic.

**C1. How important is it for you to have the identifying information for your implanted device?**

***Probes***

- Why or why not?
- Did you ask for this information when you had your device implanted?
- Did you receive a wallet card for your device?

**C2. Did you know that your implanted device has a UDI?**

***Probes***

- If so, do you have the UDI for your implanted device stored somewhere?
- If yes, where do you have it – a wallet card? Written down someplace?

**C3. What are your thoughts about giving the UDI to patients when they have a device implanted?**

***Probes***

- Who do you think should give the UDI to patients? (doctor/nurse, manufacturer, other)
- How would you like to have it? (card, document, electronically: patient portal, other app)

**D. STORING UDI (10 minutes)**

Next, we are going to talk about storing or documenting the UDI for your implanted device.

**D1. What are your thoughts about requiring that the UDI be stored or documented when an implanted device is put into a patient?**

**D2. Where do you think the UDI should be documented when a patient has their procedure?**

***Probes***

- In your electronic health record?
- Other places?

**D3. Who do you think has responsibility to have the record of the UDI for your implanted device?**

***Probes***

- If doctor or hospital, what would happen if they are no longer easily available to you, such as the doctor retired or you moved?
- If self, have you requested this information?

**E. SHARING UDI (15 minutes)**

Next, we are going to talk about sharing the UDI of your implanted device

**E1. What are your thoughts about the UDI being included on the insurance claim form that gets sent to the insurance company when patients get implanted devices?**

**E2. What are your thoughts about your UDI being shared with:**

***Probes***

**Probe benefits & concerns for each; Would it be ok to share information on the implanted device but no personal information?**

- Doctors that may provide you medical care
- Hospitals where you receive medical care
- A national database of information on medical devices
- The US Food and Drug Administration or FDA, the government agency that is in charge of medical device safety for the US
- The manufacturer of your implanted device

**F. USING UDI (15 minutes)**

Next, we are going to talk about different ways UDIs could be used and would like your feedback.

**F1. If a record of your UDI was kept in your electronic health record or by your insurance company, it could be used to quickly identify you if there is a problem with a device. For example, if the device is recalled**.

***Probes***

- What are your thoughts about this?
- Who do you think should notify you if there is a problem with your device?
  - Did your doctor given you information about who would notify you if your implant has a problem or is recalled?
- The FDA is in charge of determining recalls in the US. Does this change your feelings about the UDI of your implant being shared with the FDA?

**F2. If a record of your UDI was kept in your electronic health record or by your insurance company, it could be used to help your doctor provide care to you. Examples include knowing if you can use an MRI machine with your particular device or knowing what type of implanted device you have in case of an emergency or redo procedure**.

***Probes***

- What are your thoughts about his?
- Does this impact who you want to know the UDI of your implanted device or where it is documented?

**F3. UDIs****could be used in research. For example, data from lots of individual patients getting a particular device could be brought together in one big research dataset to study the device. This data could come from electronic health records, national databases of information on medical devices, or insurance claims**.

***Probes***

- What are your thoughts about this?
- What are research topics or ideas you would like to see about your device?
- Does this change your previous feelings about documenting and sharing your UDI?

**F4. If the UDI was shared with the manufacturer when you had a device implanted, UDIs could be used to help manufacturers develop better medical devices. For example, information collected from many patients on a particular device could indicate a problem**

***Probes***

- What are your thoughts about this?
- Does this change your previous feelings about sharing your UDI with the manufacturer of your implanted device?

**G ‐ Final Thoughts (10 minutes)**

**G1. What is the most important thing you learned today?**

**G2. Are there ways you want to be further involved with this area?**

**Thank you very much for your time this evening and your willingness to share your thoughts with us. As mentioned earlier, if you think of anything else you want to add please contact us to share**.
